# Comparison of Thunderstorm Simulations from WRF-NMM and WRF-ARW Models over East Indian Region

**DOI:** 10.1100/2012/951870

**Published:** 2012-05-02

**Authors:** A. J. Litta, Sumam Mary Ididcula, U. C. Mohanty, S. Kiran Prasad

**Affiliations:** ^1^Department of Computer Science, Cochin University of Science and Technology, Cochin, Kerala 682 022, India; ^2^Centre for Atmospheric Sciences, Indian Institute of Technology Delhi, Hauz Khas, New Delhi 110016, India

## Abstract

The thunderstorms are typical mesoscale systems dominated by intense convection. Mesoscale models are essential for the accurate prediction of such high-impact weather events. In the present study, an attempt has been made to compare the simulated results of three thunderstorm events using NMM and ARW model core of WRF system and validated the model results with observations. Both models performed well in capturing stability indices which are indicators of severe convective activity. Comparison of model-simulated radar reflectivity imageries with observations revealed that NMM model has simulated well the propagation of the squall line, while the squall line movement was slow in ARW. From the model-simulated spatial plots of cloud top temperature, we can see that NMM model has better captured the genesis, intensification, and propagation of thunder squall than ARW model. The statistical analysis of rainfall indicates the better performance of NMM than ARW. Comparison of model-simulated thunderstorm affected parameters with that of the observed showed that NMM has performed better than ARW in capturing the sharp rise in humidity and drop in temperature. This suggests that NMM model has the potential to provide unique and valuable information for severe thunderstorm forecasters over east Indian region.

## 1. Introduction

Thunderstorm, resulting from vigorous convective activity, is one of the most spectacular weather phenomena in the atmosphere. Northeastern part of Indian region (20°N to 24°N latitude, 85°E to 93°E longitude) experiences thunderstorms at higher frequency during premonsoon months (March–May), when the atmosphere is highly unstable because of high temperatures prevailing at lower levels. These storms predominantly come from the northwest and hence called Nor'wester, locally named as “Kal-baishakhi” [1], though they can come from other directions as well. They are often associated with moderate/severe squalls achieving a speed in the range of 130–150 km/hr, which may even reach tornadic violence causing considerable damage to property and loss of life. Such severe weather systems affect the crops and lives on the ground and aviation aloft [2]. Naturally, it has immense economic and societal impact on human existence. The associated large hailstones, high wind gust, and heavy rainfall have given the weather phenomenon a hazardous facet. Because of its propensity to harm life and property, this weather phenomenon has drawn the attention of the professional meteorologists for the last nine decades [3].

A warm, moist, and southerly low level flow from the Bay of Bengal and a cool, dry, westerly, or northwesterly upper-level flow give rise to a favorable synoptic setting for the formation of Nor'westers. Further, Nor'westers have a mesoscale structure with a very rapid development. The surface observations and radiosonde data are usually being used for forecasting Nor'westers. However, the timing and spacing of these observations are often inadequate to diagnose the evolution of preconvective conditions of Nor'westers. The understanding and prediction of these weather events is a challenge to the atmospheric scientists. STORM programme focuses a comprehensive observational and modeling study on genesis, evolution, and life cycle of intense tropical convective activities over east and northeast regions of India during premonsoon period through mesonetwork of observations and mesoscale analysis and prediction systems. As the Nor'westers also affect Bangladesh, Nepal, and Bhutan, therefore, in 2009 the field experiment was extended to cover these countries as well. A coordinated field experiment named “SAARC STORM” was conducted jointly with the 4 countries during 1–31 May 2009 [4].

Numerical modeling has made substantial advances in the modeling of convective clouds and mesoscale convective systems [5]. Many mesoscale models like MM5, WRF-ARW (advanced research WRF), WRF-NMM (nonhydrostatic mesoscale model), ARPS (advanced regional prediction system) and RAMS (regional atmospheric modeling system) have been in operational use for forecasting thunderstorms at many places in the world. A basic characteristic of these models is that their governing equations are nonhydrostatic since the vertical and horizontal scales of convection are similar. Such models are also necessary for explicitly resolving gravity waves triggered by clouds. Presently, mesoscale models having a resolution less than 9 km are also available for the simulation and prediction of regional weather systems. These models can be used for a variety of applications including simulation and prediction of heavy rainfall, severe thunderstorms, and tropical cyclones [6–8]. Thunderstorm forecasting is one of the most difficult tasks in weather prediction, due to their rather small spatial and temporal extension [9].

The understanding of the dynamical/physical mechanisms of thunderstorms is essential for improving the forecast of these systems. One of the ways to understand the physics and dynamics of these severe thunderstorms is to simulate these systems with the help of mesoscale models. A number of studies have been carried out [10, 11] to simulate thunderstorms for studying various dynamical and physical processes occurring within them. Accurate simulation requires knowledge about “where” and “when” storms will develop and how they will evolve. The high-resolution nonhydrostatic mesoscale models with sophisticated parameterization schemes for the important physical processes would be a very useful tool for reasonably accurate prediction of these severe thunderstorms [12]. However, mesoscale research and forecasting in India could not keep pace with developments of the post-1970 period, especially in respect of mesoscale observational techniques (Doppler weather radar (DWR), wind profilers, mesonetwork), mesoscale analysis, and mesoscale numerical weather prediction (NWP) [13]. In India, studies related to modeling of clouds are very scarce and in particular intense thunderstorm events [14]. Simulation of severe thunderstorms with high-resolution mesoscale models over east Indian region has been attempted by many Indian researchers recently [7, 15–17].

In the present study, an attempt has been made to compare the simulated results of three thunderstorm events (03 May 2009, 11 May 2009, and 15 May 2009) during SAARC STORM field experiment 2009, using WRF-NMM model developed by National Oceanic and Atmospheric Administration (NOAA)/National Centers for Environment Prediction (NCEP) and WRF-ARW modeling system developed by National Centre for Atmospheric Research (NCAR). The capacities of the WRF-NMM and WRF-ARW model in retrieving precipitation fields over east Indian region during three severe thunderstorm events were analyzed, by comparing the outputs of the models with ground observations. A quantitative verification of the results was performed with classical statistics parameters, namely, mean absolute error (MAE), root mean square error (RMSE), and correlation coefficient (CC). The temporal variations of temperature and relative humidity, which are useful for occurrence and intensity of the severe thunderstorms, are evaluated and validated the model results with observations. The model-simulated radar reflectivity and cloud top temperature were compared with the Kolkata DWR and Kalpana satellite imageries, to verify whether the models were able to simulate the genesis, intensification, and propagation of these thunder squalls. This study is presented in the following manner.  Section 2 presents the case description of all three cases taken up in the present study.  Section 3 presents the description of numerical model and configurations. The results and discussion are described in Section 4 and the conclusions in Section 5. 

## 2. Case Description

 For the present study, three severe thunderstorm cases during SAARC STORM field experiment 2009 have been taken, and the description of each case is as follows. 

**Figure 1:**
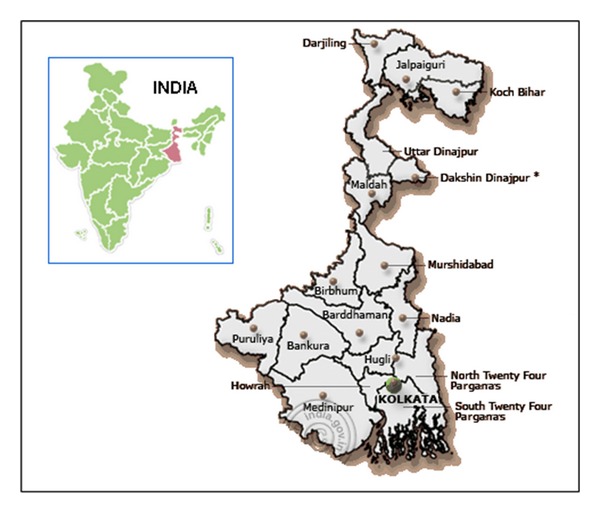
The geographical location of Kolkata in West Bengal (region of study).

Case 1 was a severe thunderstorm, which was reported on 03 May 2009 over Kolkata (Figure 1) with a maximum speed of 61.2 kmph lasting for a few minutes. This intense convective event produced 31.4 mm rainfall over Kolkata. In the synoptic charts at 0000 UTC, a low-pressure area was found at the surface over north Chattisgarh and adjoining Jharkhand, and a trough from this extending southward up to interior Tamilnadu across Andhra Pradesh is found. At 1.5 km above sea level (a.s.l), cyclonic circulation is seen over west Uttar Pradesh, and a trough from this extends southeastwards up to south peninsula across east Madhya Pradesh and Andhra Pradesh. No significant trough in midtroposphere. No subtropical westerly jet maxima were seen over the region. A few places recorded moderate rainfall over Gangetic West Bengal (GWB) and isolated rainfall over Orissa, Chattisgarh, and Bihar. Bankura recorded 24.9 mm and Sriniketan 38.2 mm of rainfall. 

Case 2 was a severe thunderstorm, which was reported on 11 May 2009 over Kolkata with squally winds of the order of 87 kmph. Rainfall of 33.3 mm was reported over Kolkata. The synoptic charts show a trough at sea level chart from east Uttar Pradesh to north Tamilnadu across east Madhya Pradesh and Andhra Pradesh. Cyclonic circulation in lower levels is found over Bihar and neighborhood. Trough from this extends up to extreme south peninsula across Chattisgarh, Telangana, and Rayalaseema. Another cyclonic circulation was existed over Arunachal Pradesh and adjoining Assam and Meghalaya. A trough from Arunachal Pradesh to northwest Bay of Bengal was found in middle troposphere. Subtropical westerly jet maxima were found over the region. Light-to-moderate rain occurred at few places over Orissa and GWB with Midnapore and Alipore reporting 17.8 mm and 21.9 mm, respectively. 

Case 3 was a severe thunderstorm, which was reported on 15 May 2009. A squall passed over Kolkata at 1230 UTC on 15 May 2009 with a maximum speed of 68.4 kmph. This intense convective event produced 16.9 mm rainfall over Kolkata. The synoptic charts show a trough at sea level from east Madhya Pradesh to south coastal Tamilnadu across Telangana and another trough to northeast Bay of Bengal across Orissa. Cyclonic circulation seen in lower levels over West Uttar Pradesh and a trough from this extends up to coastal Andhra Pradesh across Vidarbha with embedded cyclonic circulation over Telangana. Trough in midtroposphere is found from Arunachal Pradesh to north Bay of Bengal. Subtropical westerly jet maxima were found over the region. A few places of GWB recorded moderate rainfall and isolated rainfall over Orissa and Bihar. Bankura recorded 34.0 mm and Midnapore 51.6 mm of rainfall [4]. 

The common feature in the synoptic situation in all the cases was a trough, or a low pressure area was observed at the surface extending from Uttar Pradesh/Chattisgarh to Tamilnadu along Andhra Pradesh, with cyclonic circulations at 1.5 km a.s.l over Uttar Pradesh/Bihar. A trough in the mid troposphere was found in cases 2 and 3 (11, May, 2009 and 15, May, 2009) which was absent in the first case. In the upper levels the westerly jet maxima was providing good amount divergence in Cases 2 and 3, but was not found in case 1. Moisture incursion was found in the lower levels in all the three cases with moist southerly/southwesterly winds from Bay of Bengal sweeping the domain. In view of the above environmental settings, it can be further seen that although there was enough moisture incursion in the first case, the convective system developed on the day was comparatively weak which displayed weak echoes. This can be attributed to nonsupportive synoptic situation in the mid and high levels, where as in the other two cases the synoptic situation was favorable both in the lower and higher levels producing stronger echoes as were witnessed in DWR images (Figures 4, 7, and 10). The direction of movement of the squall lines generated over the western part of the domain over Jharkhand was southwesterly, and the ones initiated over northwest Bangladesh were southerly. This feature of movement of the squall lines was similar in all the three cases. So the cases basically differ in convection getting initiated under weaker and stronger synoptic situation. 

## 3. Numerical Model

 NMM and ARW modeling systems were used in this study to perform cloud-resolving simulation of thunderstorm events that occurred over east Indian region during the field experiment of SAARC STORM programme 2009. Several studies related to the simulation of severe thunderstorm events using NMM model have been performed worldwide [12, 18, 19]. Researches related to comparison of impacts of ARW and NMM mesoscale dynamic cores over the US have been performed [20–22]. This study is the first attempt in India to compare ARW and NMM models in the same WRF framework for the simulation of thunderstorm events. 

NMM runs are initialized through the same basic mechanism as the ARW runs: the WRF preprocessing system (WPS) reads GRIB data from an initializing model and interpolates it onto the target WRF domain grid. However, the functionality of the WPS had to be expanded to handle the horizontal staggering, map projection, and vertical coordinate used by the NMM, as each is distinct from its ARW counterpart. The NMM is a fully compressible, nonhydrostatic mesoscale model with a hydrostatic option. The model uses a terrain following hybrid sigma-pressure vertical coordinate. NMM model surfaces are terrain-following sigma surfaces near the ground, purely isobaric above a prescribed pressure value (typically about 420 hPa), and relax from terrain following to isobaric over the intervening depth. Further details of the vertical coordinate can be found in [23], while ARW model's vertical coordinate is a terrain-following hydrostatic pressure coordinate. 

 Another key difference between the NMM and ARW relevant to model initialization is the use of a rotated latitude-longitude grid in the NMM. The simplicity of a latitude-longitude grid is made applicable over the entire globe by rotating the earth's latitude-longitude grid such that the equator and prime meridian intersect at the center of the NMM's computational grid. This rotation minimizes the convergence of meridians, keeping the true horizontal scale relatively uniform over the domain. The grid staggering used in NMM model is the Arakawa E-grid, where both wind components are collocated on the same grid point offset from the associated mass point. Rationale for selecting an E-grid over the more widely used Arakawa C grid is discussed elsewhere [24]. ARW model grid staggering is the Arakawa C-grid. 

NMM model uses a forward-backward scheme for horizontally propagating fast waves, implicit scheme for vertically propagating sound waves, Adams-Bashforth scheme for horizontal advection, and Crank-Nicholson scheme for vertical advection. The same time step is used for all terms. The dynamics conserve a number of first- and second-order quantities including energy and enstrophy [25], while ARW model uses higher-order numerics. This includes the Runge-Kutta 2nd- and 3rd-order time integration schemes and 2nd- to 6th-order advection schemes in both horizontal and vertical directions. It uses a time-split small step for acoustic and gravity-wave modes. The dynamics conserves scalar variables. Both models support a variety of capabilities, which include real-data simulations, full physics options, nonhydrostatic and hydrostatic (runtime option), one-way static nesting, and applications ranging from meters to thousands of kilometers. 

In the present study, both ARW and NMM models were integrated for a period of 24 hours starting from 0000 UTC of each day and ending at 0000 UTC of the following day. Boundary and initial conditions for both models are from the high-resolution global final (FNL) analyses data set of NCEP with 1.0^0^ × 1.0^0^ lat/lon grids. Both models thus have a common starting point and avoid a potential source of difference. A single domain was configured with 3 km horizontal spatial resolution, which is reasonable in capturing the mesoscale cloud clusters. The domain covers 84.5°E to 92.5°E and 19.5°N to 27.5°N, and the grids are centered at 88.5°E, 23.5°N. Both NMM and ARW domains are configured with vertical structure of 38 unequally spaced sigma (nondimensional pressure) levels. In this study, we chose the same physics options for both the ARW and NMM simulations. Both models used Geophysical Fluid Dynamics Laboratory (GFDL) for radiation [26], Mellor-Yamada-Janjic (MYJ) scheme [27] for planetary boundary layer, Ferrier scheme [28] for microphysics, Janjic similarity scheme for surface layer [29], Noah land surface scheme for land surface [30], and Grell-Devenyi cloud ensemble scheme [31] for cumulus parameterization. All the above schemes are well tested for NMM and ARW models.  Table 1 shows the model configuration of the present study. 

Output from each model is postprocessed to bring them back to a common format that enables direct comparison. The NCEP WRF postprocessor (WPP) vertically interpolates output from each model onto isobaric surfaces, diagnoses various fields not directly computed by the models, and generates a GRIB file on the model's native projection (rotated latitude longitude for the NMM and mercator for the ARW). NCEP's “product generator” horizontally interpolates the data from each model onto a common grid used for visualization and verification. The hourly observations of AWS data, DWR imageries over Kolkata, Kalpana satellite imageries and rain gauge observations collected during the SAARC STORM field experiment 2009 are used in this present study for model validation. 

## 4. Results and Discussion

A number of meteorological conditions are required for convection to start. These conditions are instability, a sufficiently deep humid layer in the lower and middle troposphere, and an updraft, which are needed to initiate convection, since the updrafts related to processes on a synoptic scale are too slow to lift the air to the level of free convection (LFC). The formation of thunderstorms is an interaction between these conditions on different scales. Convective systems depend primarily on large-scale processes that develop an adequate thermodynamic structure, whereas processes on a mesoscale act basically at the beginning of the convective phenomenon [32, 33]. In this paper, we are analyzing some of the severe thunderstorm-affected parameters by comparing NMM and ARW model for three severe thunderstorm cases over east Indian region. 

### 4.1. Analysis of Stability Indices

Stability indices have been a cornerstone in the forecasting of convection for many decades and often are used in the research literature as well. These indices are very helpful in predicting the severe weather events. The indices are having critical values and above these critical values, we can say that there is possibility of the severe convection. Studies on the efficiency of different stability indices for the thunderstorm prediction have been made by several authors [34–36]. Advection of warm air in the lower levels and cold air in the upper levels (generally associated with deep troughs in upper tropospheric westerlies) increases the conditional instability in the atmosphere and favor outbreak of severe thunderstorms [37, 38]. 

The introduction of an index by Showalter [39] represents a watershed moment, beyond which we have seen a steady proliferation of indices, which are lifted index, SWEAT, K index, total totals index, CAPE, and so forth. Many of these indices are keyed to mandatory pressure levels, with Showalter's prototype, for example, being tied exclusively to 850 and 500 hPa. Convective available potential energy (CAPE) is perhaps the index least dependent on mandatory pressure levels, since it involves integration between levels of some physical significance (the LFC and the LNB). CAPE represents the amount of buoyant energy available to accelerate a parcel vertically, and a CAPE value greater than 1500 Jkg^−1^ is suggested by Rasmussen and Wilhelmson [40] as being necessary for super cells to form. Lifted index (LI) measures the difference between a parcel's temperatures compared with the environmental temperature at 500 hPa, after the parcel has been lifted from the lifting condensation level [41]. The LI is proved useful for indicating the likelihood of severe thunderstorms. The chances of a severe thunderstorm are best when the LI is less than or equal to −3. This is because air rising in these situations is much warmer than its surroundings and can accelerate rapidly and create tall and violent thunderstorms. 

 The K index (KI) is a combination of the vertical totals (VT) and lower tropospheric moisture characteristics. The VT is the temperature difference between 850 and 500 hPa, while the moisture parameters are the 850 hPa dew point and 700 hPa dew point depression. The KI has proved useful in indicating the probability of severe thunderstorms. As the KI increases, so does the probability of having a severe thunderstorm [41]. Miller [42] introduced the total totals index (TTI) for identifying areas of potential thunderstorm development. It accounts for both static stability and the presence of 850 hPa moisture. A TTI of greater than 44 indicates favorable conditions for development of severe thunderstorms [41]. The question is how effective these are when employed as thunderstorm predictors, that is, used for “thundery” or “non thundery” forecasts according to a certain threshold value. Usually convective indices are employed to alert the meteorologist on thunderstorm occurrence or nonoccurrence. Often a certain threshold value is defined above (below) which the possibility of thunderstorms is considered.  Table 2 shows the different stability indices and their critical values for severe thunderstorms. It should be noted that some of these indices are best suited for forecasting during certain conditions. For example, the KI is optimal for predicting air mass thunderstorms [40]. 

In the present study, an attempt is made to examine different stability indices obtained from NMM and ARW model in three thunderstorm days during SAARC STORM field experiment 2009 at 0000 UTC and 1200 UTC over Kolkata (22.52°N, 88.37°E). FNL-analyzed data has been used for the validation of model-simulated stability indices.  Table 3 shows the intercomparison of FNL-analyzed and model-simulated stability indices over Kolkata at 0000 UTC and 1200 UTC. The NMM- and ARW-model-simulated CAPE values are high and greater than the critical level at 0000 UTC and 1200 UTC of these three thunderstorm events, which is a favorable condition for severe thunderstorms. The model-simulated CAPE values of 03, May, 2009 at 1200 UTC are close to the FNL-analyzed values (Table 3). It is also seen that except on 15, May, 2009, where the FNL value of CAPE is less than that of the model-simulated values, on all other days the FNL analyzed and simulated CAPE values are greater than 2000 J/kg. On 15, May, 2009, the NMM- and ARW-model-simulated CAPE values were 2993 J/kg and 3554 J/kg which are far greater than the FNL-analyzed CAPE value of 656 J/kg. The mean of simulated CAPE values at 1200 UTC is found to be much larger than the FNL-analyzed values indicating that the models tend to simulate large CAPE values at 1200 UTC, when commonly thunderstorms occur. The mean of simulated LI values of both the models at 0000 UTC is nearly the same and is less than the critical level and a similar trend as that of CAPE values at 1200 UTC, where the difference is large between the FNL-analyzed and simulated values. The mean of simulated TTI at 0000 UTC and 1200 UTC shows a high value (more than 48), which is a favorable for severe thunderstorm occurrence and is in exact match with the FNL-derived values at 0000 UTC. The mean of simulated KI values of both the models at 0000 UTC is in good agreement with that of FNL-derived KI value, but the values at 1200 UTC are far less than the FNL-derived value. But NMM values are more matching with the critical value (>33) than ARW required for thunderstorm occurrence. Examination of all the model-simulated stability indices for each thunderstorm day clearly indicated that both the models have done well in capturing the instability of the atmosphere at 0000 UTC and 1200 UTC for the occurrence of a severe thunderstorm. Thus, model-simulated thermodynamic structure over Kolkata becomes conducive for a thunderstorm occurrence. 

### 4.2. Analysis of Precipitation

Precipitation is recognized as one of the most difficult parameters to forecast in numerical weather prediction [43]. Most of the thunderstorms produce heavy rainfall during their lifecycle of 1–3 hours. The precipitation analyzed in the present paper was 24 h accumulated rainfall for three severe thunderstorm days by taking 6 rain gauge stations for each case. The precipitation is accumulated for up to 24 h, starting from 0000 UTC of each day and ending at 0000 UTC of the following day. Comparison of modeled precipitation with rain gauge station observations for all three thunderstorm days is given in Table 4. Both the models have well simulated the rainfall amount with NMM performing better than ARW as indicated in the table. It can also be seen from the values in Table 4 that NMM model had predicted the rainfall amount better than ARW on 03, May, 2009 at the stations Dum Dum, Bankura, Basirhat and Balasore while ARW's predictions were better at Sriniketan and Jamshedpur. The average rainfall from all these six rain gause stations is also given in Table 4 for all thunderstorm cases. NMM simulated average rainfall from all six stations are more than ARW model. On 11 May 2009, NMM model has done better than ARW model in simulating rainfall at all the 6 stations. ARW model simulated very less rainfall in 5 stations and overpredicted the rainfall amount at Kharagpur. NMM-simulated average rainfall on this day is very good as compared to ARW. On 15, May, 2009, both the models have well simulated the rainfall amount with NMM performing better than ARW as indicated in Table 4. Although ARW has done well occasionally in simulation of rainfall at rain gauge stations, the overall performance was better with NMM model. In order to analyze the modeled precipitation, statistical analysis has been done by calculating the CC, RMSE, and MAE which is given in Table 5. All three statistical parameters are calculated by taking precipitation value of six rain gauge stations for three thunderstorm cases together. NMM model's superior performance is witnessed with high-correlation coefficient of 0.565, better than that of ARW. Further, it can be seen that RMSE, MAE of NMM are less than that of ARW indicating better efficiency of NMM model in predicting rainfall at different stations. The statistical analysis shows that NMM model's predicted rainfall amounts are closer to that of the observed in comparison with that of ARW. So NMM model has outperformed ARW in rainfall prediction and is superior to the two models. 

### 4.3. Analysis of Surface Relative Humidity and Temperature

**Figure 2:**
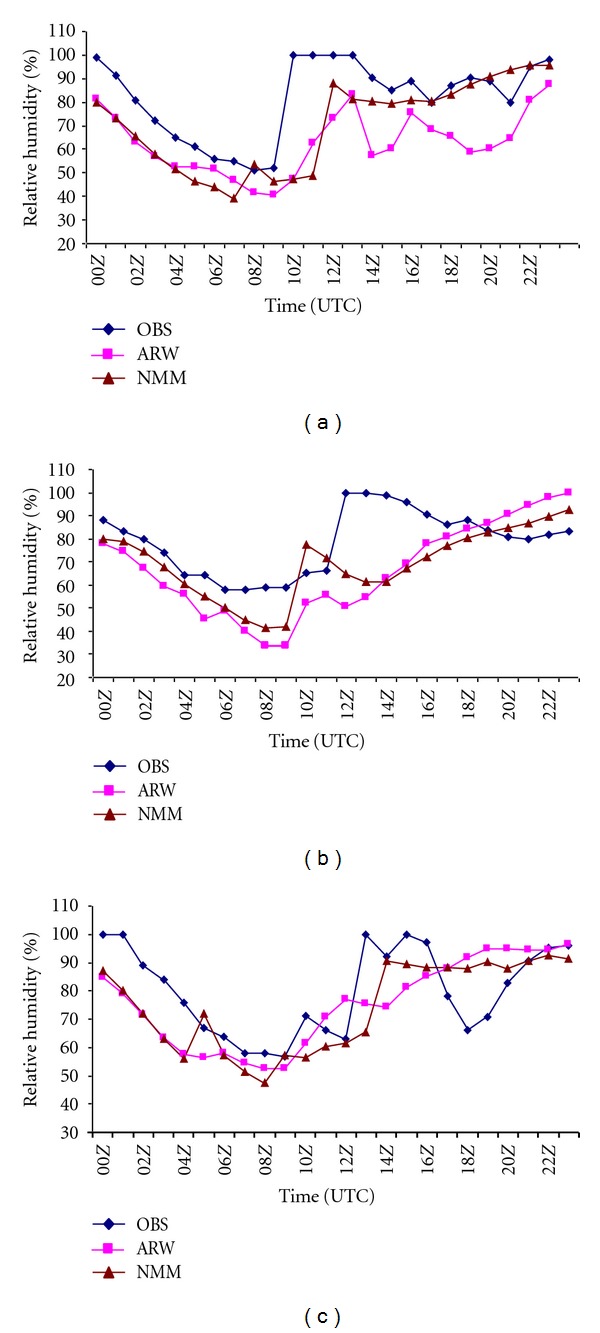
Inter comparison of NMM and ARW model simulated and observed diurnal variation of surface relative humidity (%) over Kolkata on (a) 03 May 2009 (b) 11 May 2011 (c) 15 May 2011.

Surface parameters play a significant role in the genesis, whereas the strength of the upper air pull is required to asses the growth of the thunderstorm [44]. Relative humidity at surface level has been taken into account, as it is an essential factor in intense convection. Storm days require a sufficiently humid and deep layer in the lower and middle atmosphere [33].  Figure 2 shows the intercomparison of observed and model-simulated relative humidity (%) using NMM and ARW model over Kolkata valid for 03, May, 2009, 11, May, 2009 and 15, May, 2009 at 0000 UTC to next day at 0000 UTC. The observed relative humidity for 03, May, 2009 (Figure 2(a)) values peaked from 52% to 100% (48%) at 1000 UTC, whereas NMM model showed a sharp rise from around 49% to 88% (39%) at 1200 UTC, which is two hours later than that of the observed. ARW was not able to capture the sharp rise of relative humidity during the thunderstorm hour as in the NMM model. In the second case (Figure 2(b)), observed relative humidity showed a rise from 66% to 100% (34%) at 1200 UTC, whereas NMM simulation shows a rise from 42% to 77% (35%) at 1000 UTC, which is two hours prior than that of the observed. ARW model simulation shows an increase from 34% to 52% (18%). A sudden increase of 35% has been captured by NMM model as in the observed rise of 34%. ARW model is able to capture the rise with less intensity. In the third case (Figure 2(c)), observed relative humidity peaked from 63% to 100% (37%) at 1300 UTC, whereas NMM model shows a sharp rise from 65% to 91% (26%) at 1400 UTC, which is one hour later than that of the observed. In this thunderstorm case, also ARW model was not able to capture the sharp rise of relative humidity during the thunderstorm hour as in the NMM model. For all the thunderstorm cases (Figure 2), NMM model has captured the sudden rise of relative humidity values during the model-simulated thunderstorm hour as in the observations. 

**Figure 3:**
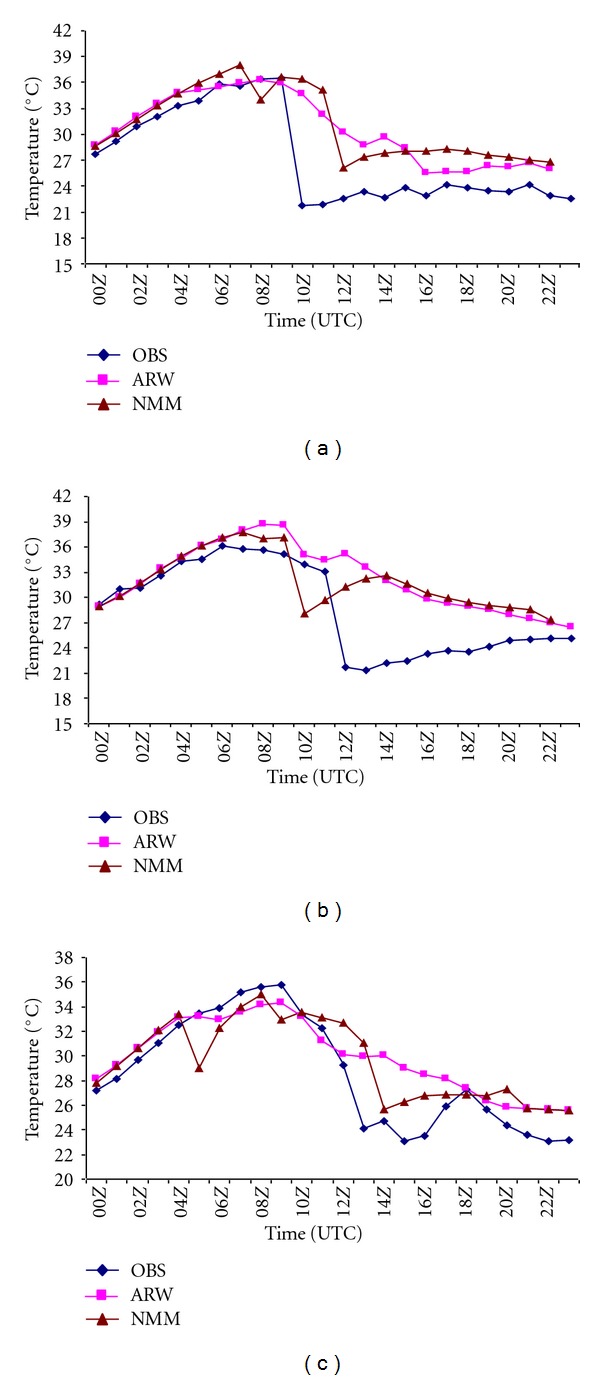
Inter, comparison of NMM- and ARW-model-simulated and observed diurnal variation of surface temperature (°C) over Kolkata on (a) 03, May, 2009 (b) 11, May, 2011 and (c) 15, May, 2011.

Surface temperature is useful parameter in forecasting the likelihood occurrence of a thunderstorm [45].  Figure 3 shows the intercomparison of observed and model-simulated temperature (°C) using NMM and ARW model over Kolkata valid for 03, May, 2009, 11, May, 2009, and 15, May, 2009 at 0000 UTC to next day at 0000 UTC. The observed temperature (Figure 3(a)) showed a sudden fall from 36.7°C to 21.7°C (15°C) at 1000 UTC, whereas NMM model showed a fall from 35.1°C to 26.1°C (9°C) at model predicted hour. For the second case (Figure 3(b)), observed temperature showed a drop from 33.1**°**C to 21.7**°**C (11.4**°**C) at 1200 UTC, whereas NMM simulation shows a drop from 37.1**°**C to 28**°**C (9.1°C) at 1000 UTC. In the third case, the observed temperature (Figure 3(c)) showed a sudden fall from 29°C to 24°C (5°C) at 1300 UTC, whereas NMM model showed a fall from 31°C to 26°C (5°C) at model predicted hour of 1400 UTC. In all three cases, ARW model failed to capture the sudden temperature fall over Kolkata as in NMM model. Comparison of the surface parameters simulated by both the models indicates the superiority of NMM model in simulating the thunderstorm over Kolkata on these severe thunderstorm cases even though one- or two-hour lead or lag exists. 

### 4.4. Analysis of Composite Radar Reflectivity

**Figure 4:**
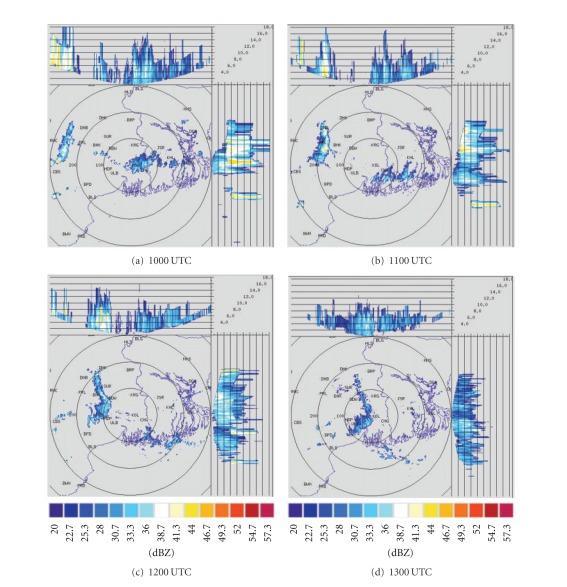
Kolkata Doppler weather radar (DWR) composite radar reflectivity (dBZ) imageries from 1000 to 1300 UTC on 03, May, 2009.

**Figure 5:**
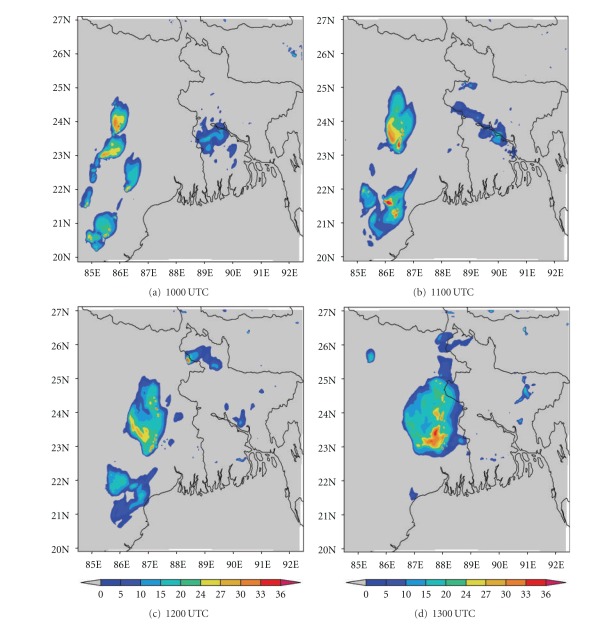
NMM-simulated composite radar reflectivity (dBZ) pictures from 1000 to 1300 UTC on 03, May, 2009.

**Figure 6:**
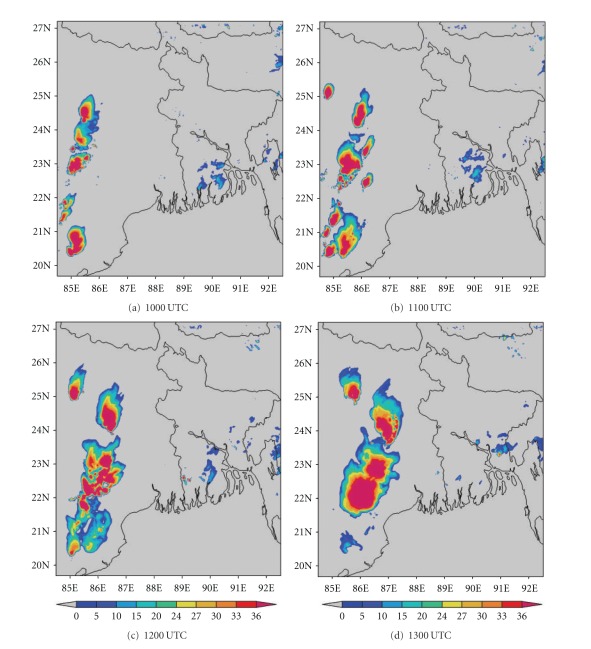
ARW-model-simulated composite radar reflectivity (dBZ) from 1000 to 1300 UTC on 03, May, 2009.

DWR is being used worldwide for the study of various severe weather phenomena like thunderstorms, hailstorms, tornados, and cyclones. In other words, it can measure how fast rain or hail is moving towards or away from the radar. From a volume scan (a series of 360-degree sweeps, each tilting a little higher than the last) forecasters can get a detailed look at structures and movements in storms close to the radar [15]. By analyzing Kolkata DWR composite radar reflectivity (dBZ) imageries, on 03, May, 2009, a strong echo was developed northwest of Kolkata (Ranchi) at 0900 UTC. This echo intensified into northsouth oriented squall line by 1000 UTC (Figure 4(a)) and gradually moved towards Kolkata at 1100 UTC (Figure 4(b)). This echo was over Kolkata at 1300 UTC (Figure 4(d)) and disappeared at 1400 UTC [4]. The use of composite radar reflectivity fields as a model output product has become increasingly popular recently as a means for display of high-resolution numerical model fields. The chief advantage of the model reflectivity product appears to be that it allows one to more easily see detailed mesoscale and near-storm scale structures capable of being simulated by finer resolution models, such as the structure of deep convection, movement of squall line, and frontal precipitation bands [21]. NMM-model-simulated composite radar reflectivity (dBZ) on 03, May, 2009 from 1000 to 1300 UTC is shown in Figure 5. By analyzing NMM-model-simulated composite radar reflectivity plots, a squall line developed northwest of Kolkata at 1000 UTC. This squall line was moving towards Kolkata at 1100 UTC and was over Kolkata at 1300 UTC as in the DWR imageries. ARW-model-simulated composite radar reflectivity (dBZ) on 03, May, 2009 from 1000 to 1300 UTC is shown in Figure 6. ARW-model-simulated composite radar reflectivity plots also show a squall line, which developed northwest of Kolkata at 1000 UTC as in NMM model. This squall line was moving towards Kolkata at 1100 UTC, but did not reach Kolkata at 1300 UTC, which indicates the slow movement of the squall line. The squall line movement and intensity were well captured by NMM than ARW. 

**Figure 7:**
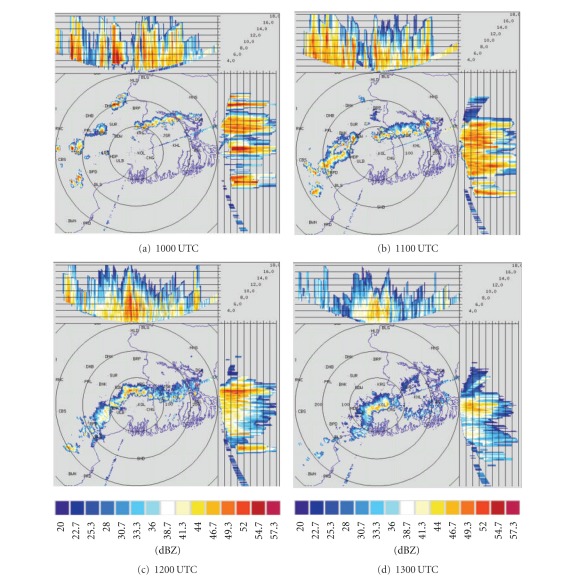
Kolkata DWR composite radar reflectivity (dBZ) imageries from 1000 to 1300 UTC on 11, May, 2009.

**Figure 8:**
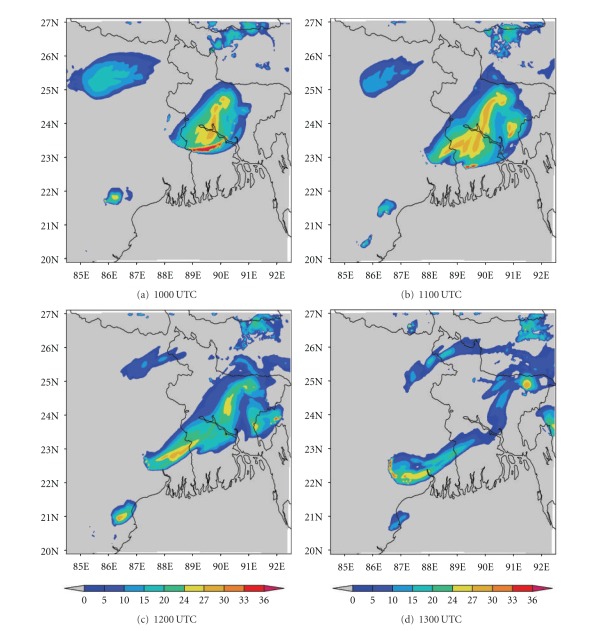
NMM-simulated composite radar reflectivity (dBZ) pictures from 1000 to 1300 UTC on 11, May, 2009.

**Figure 9:**
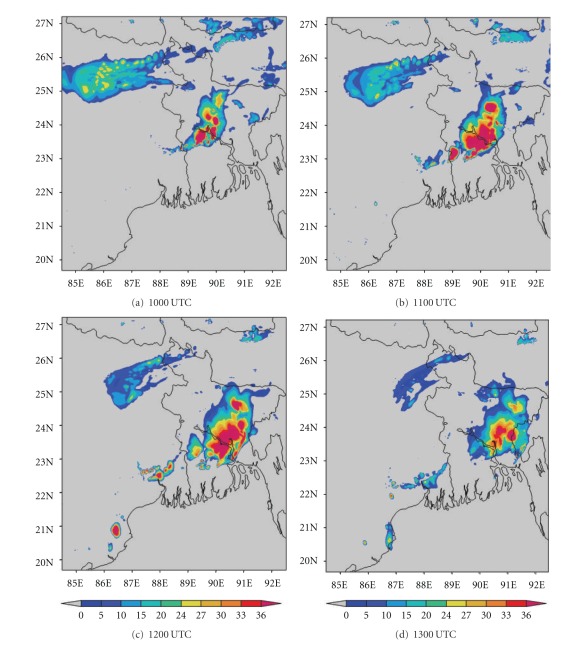
ARW-simulated composite radar reflectivity (dBZ) pictures from 1000 to 1300 UTC on 11, May, 2009.

**Figure 10:**
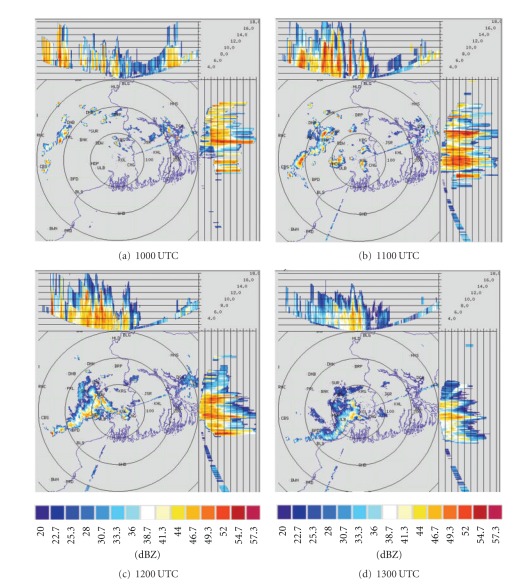
Kolkata DWR composite radar reflectivity (dBZ) imageries from 1000 to 1300 UTC on 15, May, 2009.

**Figure 11:**
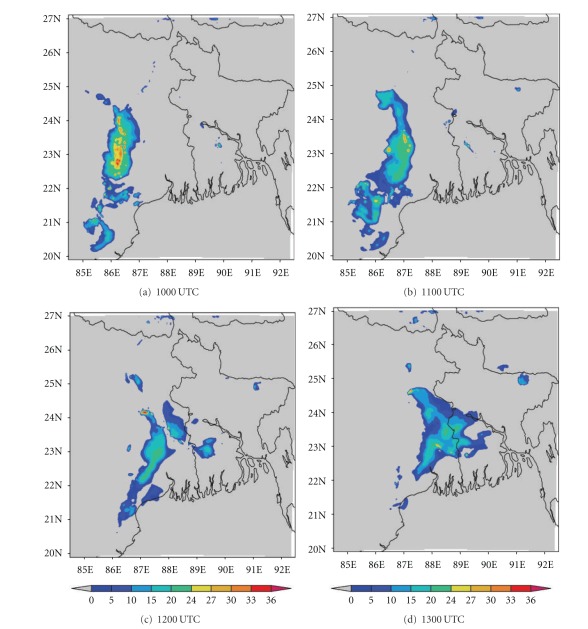
NMM-simulated composite radar reflectivity (dBZ) pictures from 1000 to 1300 UTC on 15, May, 2009.

**Figure 12:**
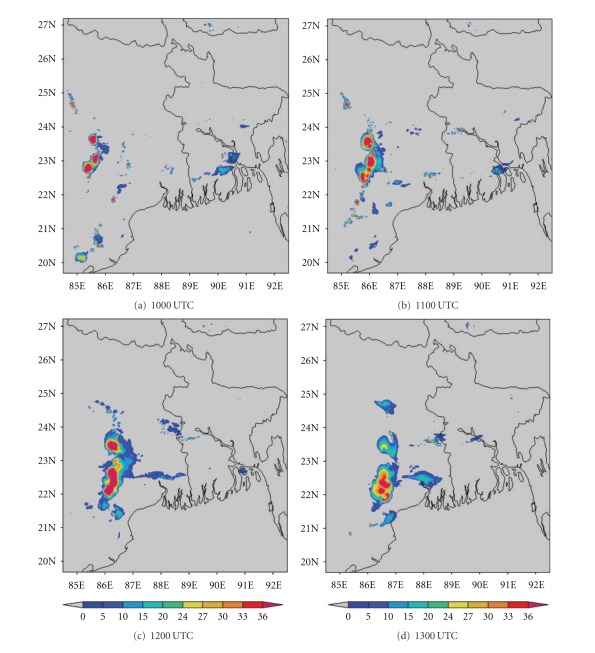
ARW-model-simulated composite radar reflectivity (dBZ) from 1000 to 1300 UTC on 15, May, 2009.

Kolkata DWR imageries from 1000 to 1300 UTC on 11, May, 2009 are given in Figure 7. By analyzing Kolkata DWR imageries of 11, May, 2009, a strong echo was developed northeast of Kolkata at 1000 UTC, which was intensified into west east-oriented squall line by 1100 UTC. Another strong echo was developed at the northwest of Kolkata at 1100 UTC. These two echoes are merged at 1200 UTC and become intensified. This echo gradually moved towards Kolkata at 1300 UTC. Both ARW and NMM models failed to capture two strong echoes in their plots. They are able to simulate one echo which was initiated from northeast of Kolkata at 1000 UTC as in observation. It was intensified and moved towards Kolkata at 1100 UTC (Figure 8). NMM model well captured this squall line movement as compared to ARW model (Figure 9) even though the magnitude of composite radar reflectivity simulated by NMM model is less. By analyzing Kolkata DWR imageries on 15, May, 2009 (Figure 10), a strong echo was developed near Purulia (PRL) at 1000 UTC, which intensified into northsouth-oriented squall line by 1100 UTC. This echo gradually moved towards Kolkata at 1200 UTC. This echo was over Kolkata at 1300 UTC and disappeared at 1500 UTC. NMM-model-simulated composite radar reflectivity on 15, May, 2009 from 1000 UTC to 1300 UTC is shown in Figure 11. By analyzing NMM-model-simulated composite radar reflectivity plots, a squall line developed northwest of Kolkata at 1000 UTC. This squall line was moving towards Kolkata at 1100 UTC and was over Kolkata at 1300 UTC as in the DWR imageries. By analyzing ARW-model-simulated composite radar reflectivity pictures (Figure 12), a squall line developed northwest of Kolkata at 1000 UTC as in NMM model. This squall line was moving towards Kolkata at 1100 UTC. This echo was not reached over Kolkata by 1300 UTC as in the DWR imageries and NMM-simulated outputs. ARW model well simulated the intensity as in the previous case. However, it is seen that the movement of the squall line was slow in ARW as compared to that of the observed. 

Simulated radar reflectivity is beholden to the fidelity of the model cloud and precipitation microphysics forecast, since it is derived directly from the hydrometeor mixing ratios. Any biases in those mixing ratios will be reflected in the simulated reflectivity field. Furthermore, a particular challenge in trying to produce a simulated reflectivity product is the diameter-to-the-sixth-power dependence of equivalent reflectivity factor. This dependence renders reflectivity highly sensitive to the largest precipitation particles present and thus renders simulated reflectivity highly sensitive not only to the precipitation mixing ratios, but to assumptions about the precipitation size distributions. It is conceivable that a model could be performing well in terms of precipitation forecast, but producing unrealistic reflectivity fields due to poor representation of the particle size distributions [21]. From the present analysis of the simulated composite radar reflectivity, we conclude that NMM model has reasonably well simulated genesis, intensification, and propagation of three severe thunderstorms during 2009 premonsoon season over east Indian region as in the DWR radar reflectivity imageries, but failed to capture the intensity as in observations for second and third thunderstorm cases. ARW model well simulated the thunderstorm initiation, while the squall line movement was slow. 

### 4.5. Analysis of Cloud Top Temperature

**Figure 13:**
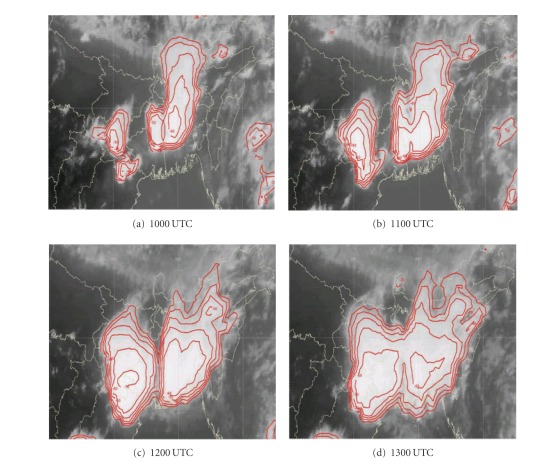
Kalpana satellite derived cloud top temperature (°C) imageries from 1000 to 1300 UTC on 03, May, 2009.

**Figure 14:**
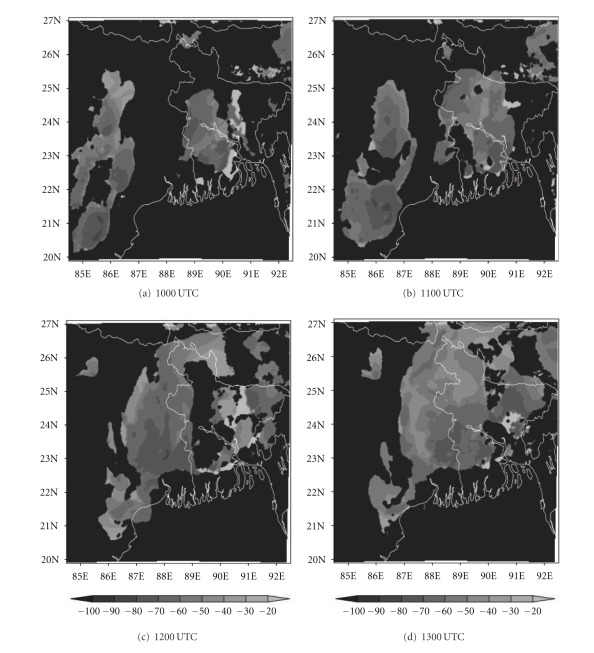
NMM-model-simulated cloud top temperature (°C) from 1000 to 1300 UTC on 03, May, 2009.

**Figure 15:**
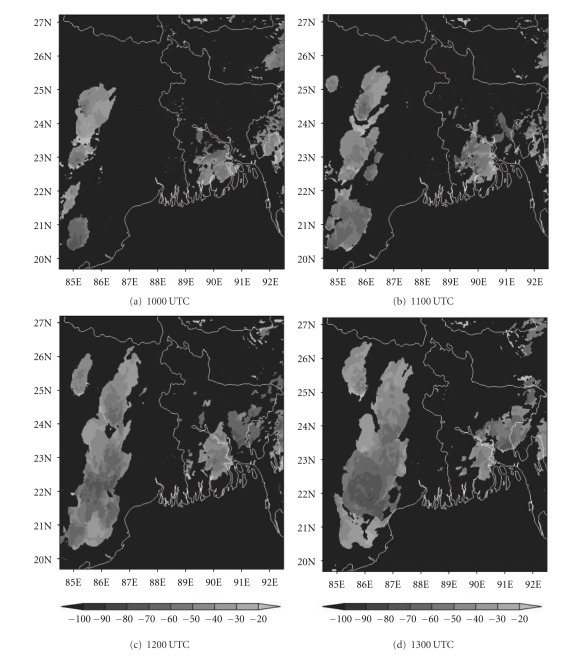
ARW-model-simulated cloud top temperature (°C) from 1000 to 1300 UTC on 03, May, 2009.

The ability to accurately forecast cloudiness is necessary in the fields of aviation. In recent years, brightness temperature and cloud top temperature derived from NWP model output have been used to demonstrate the advanced capabilities of these models for severe weather prediction [46]. In this section, we have examined the ability of NMM and ARW models to realistically simulate the cloud top temperature (CTT) over east Indian region. The comparison of Kalpana satellite-derived cloud top temperature imageries with model simulated CTT is presented here. The satellite imageries (Figure 13) of this thunderstorm case show that two convective cells developed over Bangladesh (northeast of Kolkata) and Jharkhand (northwest of Kolkata) at 1000 UTC. These cells expanded and merged over West Bengal by 1200 UTC and reached a maximum CTT of −60°C. This cell is more intensified at 1300 UTC and reached upto −70°C. The NMM-model-simulated CTT (Figure 14) also shows both convective cells over northeast and northwest of Kolkata at 1000 UTC. These cells are merged over West Bengal at 1200 UTC as in the satellite imageries. The model-simulated CTT reached upto −70°C during this cloud formation and movement. The ARW-model-simulated CTT shows the cloud cluster over northwest of Kolkata as in the NMM model (Figure 15). The ARW model failed to capture convective cell over northeast of Kolkata as in NMM and observed imageries. The movement of this cloud cluster simulated by ARW model is slow as in DWR imageries. The NMM-model-simulated CTT for other two cases also show cloud clusters over West Bengal region as in observations. But ARW model failed to represent the cloud clusters as in observations (results not shown). The convection diagnosed by the cloud top temperature from NMM model appears to be fairly representative of the structure and intensity observed in Kalpana satellite imageries. 

## 5. Conclusions

In the present study, an attempt has been made to compare the simulated results of three thunderstorm events during SAARC STORM field experiments 2009 which was initiated and conducted during the months of April and May over east and northeast regions of India, using WRF-NMM and WRF-ARW models, and it validated the model results with observations. 

Analysis of the stability indices simulated by both the models in comparison with that of FNL-derived indices clearly indicate that both the models have performed well in simulating the different thermodynamic indices such as CAPE, LI, TTI, and K-index at 1200 UTC which is very much favorable for thunderstorm occurrence. It is also seen that the models tend to overpredict CAPE and LI more at 1200 UTC as compared to FNL analysis. Comparison of model-simulated radar reflectivity with that of the observed revealed that both the models have done well in simulating the initiation of squall lines. NMM model has simulated well the propagation of the squall lines, which is in good agreement with that of the observed, while the squall line movement was slow in ARW. The NMM-model-simulated cloud top temperature appears to be fairly more representative of the structure and intensity observed in satellite imageries than ARW. 

Comparison of model-simulated thunderstorm affected parameters with that of the observed revealed that NMM has performed better than ARW in capturing the sharp rise in humidity and drop in temperature even though one- or two-hour lag or lead exists. ARW model has failed to capture the rise and drop in humidity and temperature, respectively. The precipitation forecasts have been analyzed by statistical techniques, namely CC, RMSE, and MAE. It is clearly seen from the analysis that NMM model has done better with high CC than that of the ARW and also with low RMSE and MAE. So it can be concluded that NMM model has out performed ARW in precipitation forecasts. From the above results, it can be concluded that NMM model has better capability in prediction of thunderstorms over east Indian region. This study is not conclusive, because the data used to evaluate model simulations are quite limited, and more diagnostic analysis would be required to understand why there are such differences between these two models. 

## Figures and Tables

**Table 1:** ARW and NMM model configuration.

Model	WRF-NMM	WRF-ARW
Dynamics	Nonhydrostatic	Nonhydrostatic
Horizontal resolution	3 km	3 km
Forecast length	24 hrs	24 hrs
Map projection	Rotated latitude and longitude	Mercator
Horizontal grid system	Arakawa E-grid	Arakawa C-grid
Vertical coordinate	Hybrid sigma to pressure vertical coordinate (38levels)	Terrain following sigma vertical coordinate (38 levels)
Radiation	GFDL/GFDL	GFDL/GFDL
Surface layer	Janjic scheme	Janjic scheme
Land surface	Noah land surface scheme	Noah land surface model
Cumulus	Grell-Devenyi	Grell-Devenyi
PBL parameterization	Mellor-Yamada-Janjic	Mellor-Yamada-Janjic
Microphysics	Ferrier (new eta) scheme	Ferrier (new eta) scheme

**Table 2:** The different stability indices and their critical values for severe thunderstorm.

Stability indices	Description	Critical values for severe thunderstorm
Lifted index	T_500_ − T_parcel_	<−3
K index	(T_850_ − T_500_) + Td_850_ − (T_700_ − DT_700_)	>33
Total Totals	(T_850_ + Td_850_) − 2(T_500_)	>44
Showalter index	T_500_ − T_850_	<−2
SWEAT index	12Td_850_ + 20(TT − 49) + 2f_850_ + f_500_ + 125(s + 0.2)	>250
CAPE	∫_*z*_*f*__^*z*_*n*_^ *g*(Tv_parcel_ − Tv_env_/Tv_env_)dz	>1500
CIN	∫_*z*_bottom__^*z*_top_^ *g*(Tv_parcel_ − Tv_env_/Tv_env_)*dz*	<50

**Table 3:** Comparison of NMM- and ARW-model-simulated stability indices with FNL analysis for three thunderstorm events during SAARC STORM field experiment 2009.

Stability indices	Critical level	Thunder storm cases	0000 UTC			1200 UTC		
			FNL	NMM	ARW	FNL	NMM	ARW
CAPE	>1500	3 May	2035	2947	3338	3412	3361	3583
		11 May	2959	3685	3455	2248	3932	3963
		15 May	2395	3033	3100	656	2993	3554
		**MEAN**	**2463**	**3221.7**	**3297.7**	**2105.3**	**3428.7**	**3700**

LI	<−3	3 May	−5	−7	−8	−7	−7	−8
		11 May	−9	−10	−9	−6	−10	−11
		15 May	−8	−9	−8	−2	−6	−8
		**MEAN**	**−7.3**	**−8.7**	**−8.3**	**−5**	**−7.7**	**−9**

TT	>44	3 May	48	50	49	47	49	50
		11 May	52	51	51	52	56	58
		15 May	51	50	50	46	43	47
		**MEAN**	**50.3**	**50.3**	**50**	**48.3**	**49.3**	**51.9**

KI	>33	3 May	20	29	30	42	29	26
		11 May	28	28	27	44	39	36
		15 May	35	33	34	37	29	28
		**MEAN**	**27.7**	**30**	**30.3**	**41**	**32.3**	**30**

**Table 4:** Comparison of modeled precipitation of three thunderstorm cases with rain gauge observations.

Date	Station	LAT	LONG	IMD	ARW	NMM
3-May-09	Dum Dum	22.39	88.27	31.4	6.56	23.26
	Bankura	23.13	87.04	24.9	12.62	14.73
	Basirhat	22.4	88.53	21.2	12.14	14.12
	Sriniketan	23.39	87.42	38.2	35.24	26.06
	Balasore	21.3	86.56	43.3	11.8	31.04
	Jamshedpur	22.44	86.12	35.8	32	15.49
	**MEAN**			**32.47**	**18.39**	**20.78**

11-May-09	Dum Dum	22.39	88.27	33.3	12.48	23.10
	Bankura	23.22	87.07	22	3.44	15.13
	Canning	22.25	88.67	26.4	12.87	21.00
	Basirhat	22.4	88.53	48.4	18.74	24.75
	Digha	21.83	87.8	24.4	0	10.08
	Kharagpur	22.2	87.19	16.8	19.31	11.99
	**MEAN**			**28.55**	**11.14**	**17.68**

15-May-09	Dum Dum	22.39	88.27	16.9	35.30	17.19
	Bankura	23.13	87.04	34	20.40	24.69
	Krishnagar	23.24	88.31	19.6	17.17	18.72
	Digha	21.5	87.48	21	18.4	18.39
	Midnapore	22.25	87.19	51.6	17.7	26.54
	Haldia	22.04	88.04	33.2	21.2	30.39
	**MEAN**			**29.38**	**21.69**	**22.65**

**Table 5:** Statistical analysis of modeled precipitation for three thunderstorm cases.

Statistical analysis	Description	NMM	ARW
Correlation coefficient (CC)	$\text{cc}=\sum \left(f_{i}-f\right)\left(o_{i}-o\right)/\sqrt{{\left(f_{i}-f\right)}^{2}{\left(o_{i}-o\right)}^{2}}$	0.565	0.121
Root mean square error (RMSE)	$\text{RMSE}=\sqrt{\left(1/N\right)\sum_{i=1}^{N}{\left(f_{i}-o_{i}\right)}^{2}}$	13.785	18.464
Mean absolute error (MAE)	MAE = (1/*N*)∑_*i*=1_^*N*^|*f*_*i*_ − *o*_*i*_|	10.905	15.379
